# The Immune System in Antarctic and Subantarctic Fish of the Genus Harpagifer Is Affected by the Effects of Combined Microplastics and Thermal Increase

**DOI:** 10.3390/ijms26209968

**Published:** 2025-10-13

**Authors:** Daniela P. Nualart, Pedro M. Guerreiro, Kurt Paschke, Stephen D. McCormick, Chi-Hing Christina Cheng, Luis Vargas-Chacoff

**Affiliations:** 1 Programa de Doctorado en Ciencias de la Acuicultura, Universidad Austral de Chile, Puerto Montt 5480000, Chile; 2 Centro FONDAP de Investigación en Dinámica de Ecosistemas Marinos de Altas Latitudes (IDEAL), Valdivia 5090000, Chile; kpaschke@uach.cl; 3 Instituto Milenio Biodiversidad de Ecosistemas Antárticos y Subantárticos (BASE), Universidad Austral de Chile, Valdivia 5090000, Chile; 4 Instituto de Ciencias Marinas y Limnológicas, Universidad Austral de Chile, Valdivia 5090000, Chile; 5 Centro de Ciências do Mar do Algarve (CCMAR/CIMAR LA), Universidade do Algarve, 8005-139 Faro, Portugal; pmgg@ualg.pt; 6 Instituto de Acuicultura, Universidad Austral de Chile, Puerto Montt 5480000, Chile; 7 Department of Biology, University of Massachusetts, Amherst, MA 01003, USA; mccormick@umext.umass.edu; 8 Department of Evolution, Ecology, and Behavior, University of Illinois, Urbana-Champaign, Urbana, IL 61801, USA; c-cheng@illinois.edu

**Keywords:** *Harpagifer antarcticus*, *Harpagifer bispinis*, PVC, immune response, notothenioids, climate change

## Abstract

Rising ocean temperatures due to climate change, combined with the intensification of anthropogenic activity, may lead to changes in the physiology and distribution of native species. Compounding climate stress, microplastic particles (MPs) enter the oceans through wastewater and the breakdown of macroplastics. Depending on their composition, they can be harmful and act as a vehicle for toxic substances, although their effects on native Antarctic and subantarctic species are unknown. Notothenioid fish are members of this group and are found inside and outside Antarctica, such as the Harpagifer, which has adapted to the cold and is particularly sensitive to thermal increases. Here, we aimed to evaluate the innate immune response in the head kidney, spleen, and foregut of two notothenoid fish, *Harpagifer antarcticus* and *Harpagifer bispinis*, exposed to elevated temperatures and PVC (polyvinyl chloride) microplastics. Adults from both species were collected on King George Island (Antarctica) and Punta Arenas (Chile), respectively. Specimens were assigned to a control group or exposed to a temperature increase (TI) or PVC microplastics (MPs), separately or in combination (MPs + TI). MP exposures were oral (gavage) for 24 h or aqueous (in a bath) for 24 and 48 h. Using real-time qPCR, we evaluated the relative gene expression of markers involved in the innate immune response, including *tlr2* (*toll-like receptor 2*), *tlr4* (*toll-like receptor 4*), *myd88* (*myeloid differentiation factor 88*), *nfkb* (*nuclear factor kb*), *il6* (*interleukin 6*), and *il8* (*irterleukin 8*). We found differences between treatments when *H. antarcticus* and *H. bispinis* were exposed independently to MPs or thermal increase (TI) in the experiment with a cannula, showing an up-regulation in transcripts. In contrast, a down-regulation was observed when exposed in combination to MP + TI, which looked to be tissue-dependent. However, transcripts related to innate immunity in the bath experiment increased when exposure to both stressors was combined, mostly at 48 h. These results highlight the importance of evaluating the effects of multiple stressors, both independently and in combination, and whether these species will have the capacity to adapt or survive under these conditions, especially in waters where temperature is increasing and pollution is also rising, primarily from MP-PVC, a plastic widely used in various industries and among the population.

## 1. Introduction

The South Pacific Ocean region of Chile has an area of approximately 22 million km^2^ and a volume of more than 71.8 million km^3^, representing 5.4% of the world’s oceans [1]. This area is increasingly threatened by fishing, anthropogenic pollution, tourism, and the introduction of non-native species through fish farming. An increasing concern is the accumulation of plastics and/or microplastics in the Pacific Ocean, which originates in part from plastic devices widely used in fishing and aquaculture activities [2,3,4]. Jorquera et al. (2022) [5] showed that there is a significant accumulation of fibers from polyethylene terephthalate and acrylics, the most abundant polymers in the aquaculture industry and other anthropogenic activities, in Chilean fjord sediments. They also demonstrated that the dynamics and fate of these plastics in fjords are different from what occurs in the open ocean. Plastics have also found their way into the Southern Ocean [6] and have been found in tissues of several Antarctic species spanning different trophic levels [7,8,9,10], including fish [11,12,13].

Today, plastic is the most abundant material produced and used for countless items, pervading across industries and daily life. The use of plastic continues to grow, and the resulting large quantities of non-biodegradable plastic waste accumulate worldwide—on coasts, seabed sediments [14], on beaches, in the water column [15], in wastewater effluents, surface waters, and even in ice [16,17]. Various types of microplastics (MPs) and/or pollutants are known to be ingested and accumulate in aquatic organisms [18,19] and terrestrial invertebrates [20,21]; however, the toxicity effects of different types of microplastics are not well known [22,23,24].

Microplastic particles are dispersed throughout the world’s oceans [25] and enter the ocean via wastewater and the breakdown of microplastics. The toxicological effect of MPs on a given species depends on the duration of exposure, the concentration, shape, chemical composition, and size of the particles. Smaller particles can cross biological barriers and accumulate in tissues and organs [26,27] and become vectors for chemical or biological toxicity [28]. Some of the studied effects of acute and chronic exposure to MPs in fish include early mortality, inflammatory responses, growth inhibition, reduced energy and feeding activity, abnormal behaviors, oxidative damage [29], immune dysfunction, and changes in lipid metabolism [30,31].

The innate immune response system is a rapid and nonspecific response that can respond to pathogens or other stressors, such as temperature fluctuations, infections, atmospheric variables, and pollutants [32,33,34,35]. Innate immunity constitutes the first line of defense, being crucial for the survival of fish in a hostile environment. Among the fundamental components of innate immunity are Toll-like receptors (TLRs), such as *tlr2* and *tlr4*, which can recognize specific and highly conserved structures common to pathogenic microorganisms, known as pathogen-associated molecular patterns (PAMPs), or damage-associated molecular patterns (DAMPs) [36,37], triggering a rapid immune response. These receptors are located on the surface of immune cells and recognize specific molecular structures of pathogens, triggering a series of intracellular responses to eliminate the threat. These molecular patterns activate Pattern Recognition Receptor (PRR) signaling, which ultimately leads to the transcription of antimicrobial and proinflammatory genes [38]. Through the *myd88* (Myeloid differentiation primary response 88) signaling pathway, *tlr2* and *tlr4*, in turn, activate the transcription factor *nfkb* (Nuclear Factor kappa-light-chain-enhancer of activated B cells), which regulates the expression of proinflammatory cytokines such as interleukin-6 (*il6*) and interleukin-8 (*il8*). These cytokines mediate inflammation and activate local immune responses, promoting pathogen clearance and cellular repair [39].

Thermal changes can act as physiological stressors, triggering DAMPs recognized by the TLRs, thus initiating an immune-like inflammatory response. In this sense, excessive activation of these genes could lead to chronic inflammation, compromising the health of the fish. The mechanism by which heat (or cold) stress activates innate immune pathways via TLRs, leading to inflammatory responses, warrants attention in current and future environmental contexts, particularly in stenothermal species such as Antarctic fish. Evolution in notothenioids has impacted several cellular responses to heat in chronic cold [40]. Although the TLR repertoire seems to have been conserved [41,42], these fish show differential immune responses to pathogenic cues and temperature depending on species and habitat [33,34,35,42,43].

Intertidal benthic fish (in Antarctic and subantarctic regions) may be particularly vulnerable as they face multiple stressors in habitats threatened by anthropogenic pollution and climate change. In these ecosystems, rapid warming and the increasing accumulation of plastic debris from fisheries and tourism activities co-occur, creating a combined pressure that may exceed the tolerance limits of local fauna [44]. The impact of thermal stress on fish physiology and immune function may, in turn, adversely affect their ability to mount effective defenses against additional anthropogenic threats, such as microplastics [45]. Temperature is also expected to modulate the biological impact of microplastics by altering ingestion rates, gut transit times, and metabolic activity, which in turn may affect the uptake, distribution, and immune consequences of these particles [46].

The Harpagiferidae family, which is distributed in Antarctica and the subantarctic, is represented by a single genus, Harpagifer (Richardson, 1844) [47]. Most species are associated with the subantarctic islands [48], with only one Antarctic species, *Harpagifer antarcticus* (Nybelin, 1947) [49], inhabiting the coasts of the Antarctic Peninsula. Its sister species, *Harpagifer bispinis*, is estimated to have diverged from its Antarctic counterpart between 1 and 4 million years ago [47]. Both species have sedentary habits and occupy restricted habitats, which makes them highly susceptible to rapid environmental alterations associated with climate change and human activities. The plastic pollution arising from tourism and other anthropogenic pressures in the Chilean Antarctic region exacerbates the risk of chemical contamination, pathogen introduction, and microplastic accumulation in the Southern Ocean [48]. Given their close evolutionary relationship but distinct geographic distributions, these Harpagifer sister species represent an excellent model for studying the combined impacts of warming and microplastics. In particular, both stressors are hypothesized to converge on shared innate immune pathways, including epithelial barrier integrity and the TLR/Myd88/NFkB signaling cascade [50].

This study aimed to determine the innate immune response of these two sister species by evaluating the transcription levels of *tlr2*, *tlr4*, *myd88*, *nfkb*, *il6*, and *il8* genes in their major immune organs—the head kidney, spleen, and foregut—upon experimental heat challenge and exposure to PVC microplastics in water or by oral delivery.

## 2. Results

Mortality

No mortality or behavioral changes were observed for any fish group during the experimental period.

(A) **Oral (cannula) MP delivery groups**

***Harpagifer bispinis***

**Figure 1:**
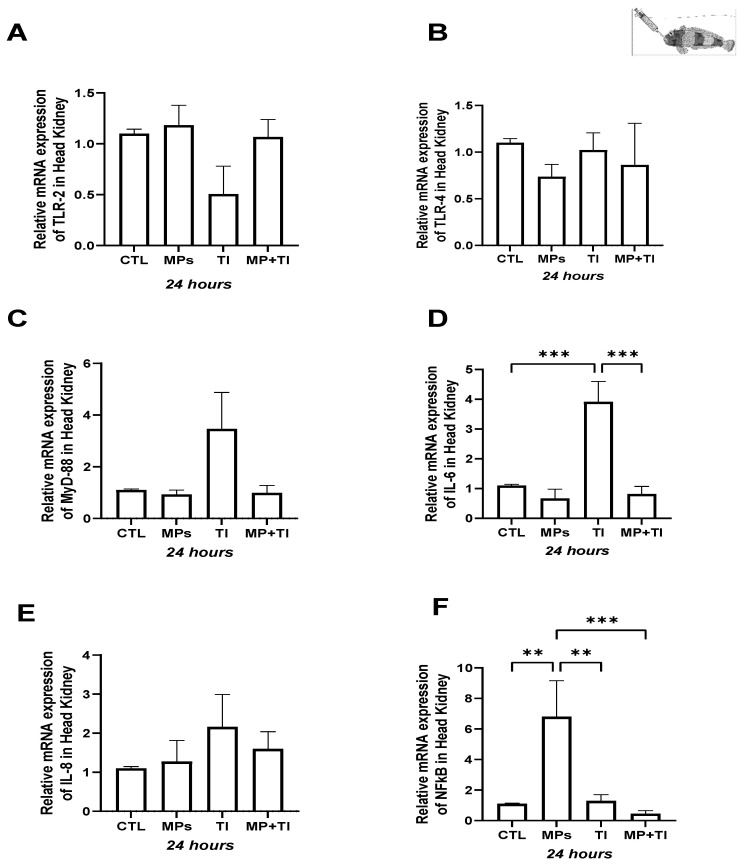
Head kidney Expression of *Harpagifer bispinis* from the cannula experiment. (**A**) *tlr2*, (**B**) *tlr4*, (**C**) *myd88*, (**D**) *il6*, (**E**) *il8* and (**F**) *nfkb*. The bars represent different treatments. CTL, control; MP, microplastics; TI, thermal increase; MP + TI, microplastics and thermal increase at 24 h. Symbols (*) over the bars indicate statistical differences between different treatments. One-way ANOVA followed by Tukey’s test (** *p* < 0.01, *** *p* < 0.001). n = 5 per sampling day/treatment.

Head kidney: Despite some variation among experimental groups, there was no apparent effect of treatments on the expression of *tlr2*, *tlr4*, *myd88*, or *il8* in the head kidney (Figure 1A–C,E). However, the largest but not statistically significant impacts were driven by increased temperature (TI). Indeed fish heated at 12 °C alone showed a significant increase (almost 4-fold) in expression of *il6* when compared with all other experimental animals, including, those at 12 °C plus MP challenged (MP + TI) (Figure 1D). However, the gene expression of *nfkb* did not respond to increased temperature, but was significantly up-regulated, six-fold in fish given oral MP, but only at the control temperature (Figure 1F).

**Figure 2:**
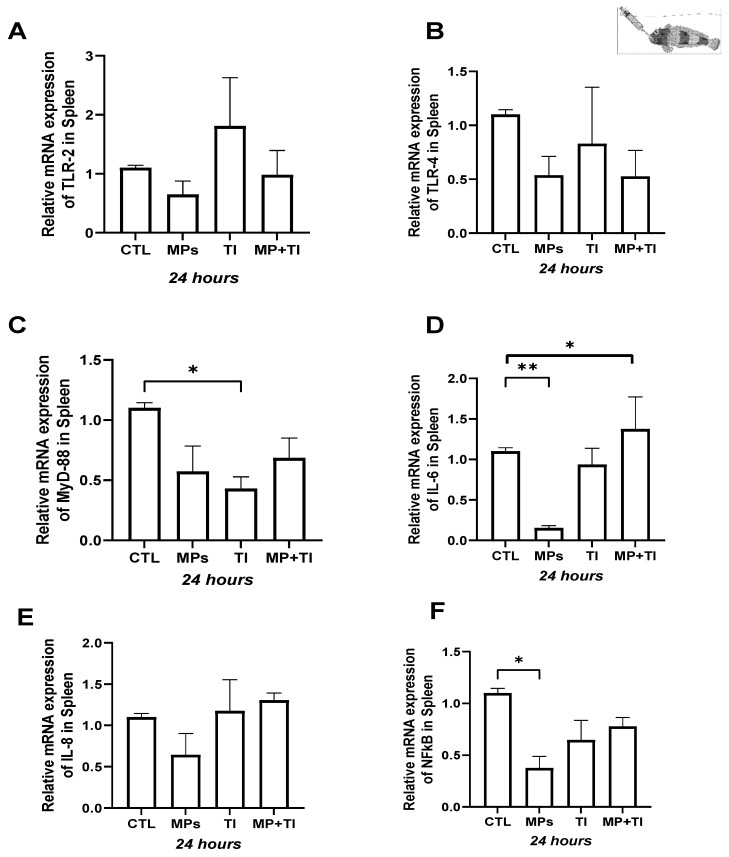
Spleen Expression of *Harpagifer bispinis* from the cannula experiment. (**A**) *tlr2*, (**B**) *tlr4*, (**C**) *myd88*, (**D**) *il6*, (**E**) *il8* and (**F**) *nfkb*. The bars represent different treatments. CTL, control; MP, microplastics; TI, thermal increase; MP + TI, microplastics and thermal increase at 24 h. Symbols (*) over the bars indicate statistical differences between different treatments. One-way ANOVA followed by Tukey’s test (* *p* < 0.05, ** *p* < 0.01). n = 5 per sampling day/treatment.

Spleen: In this organ, both *tlr2* and *tlr4* show little response to any of the challenges, with slight but not statistically significant decreases in their relative expression when exposed to MPs (Figure 2A,B). Expression of *myd88* was significantly reduced by increased temperature alone, and *il6* showed contrasting effects, as it decreased significantly with respect to the control in fish treated with MPs alone (Figure 2C,D) but showed an opposite reaction to MPs combined with increased temperature, while no effect was observed for *il8* expression (Figure 2E). Compared to the head kidney, relative expression *nfkb* in the spleen was also most affected by MPs alone but in the opposite direction (down-regulated) instead (Figure 2F).

**Figure 3:**
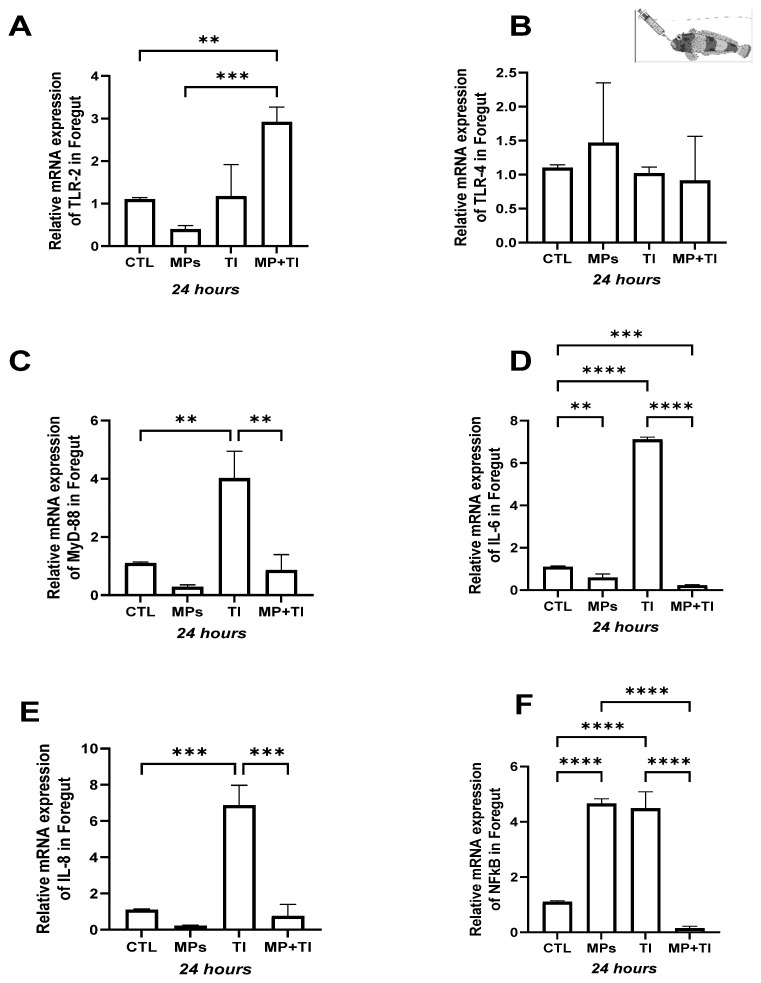
Foregut Expression of *Harpagifer bispinis* from the cannula experiment. (**A**) *tlr2*, (**B**) *tlr4*, (**C**) *myd88*, (**D**) *il6*, (**E**) *il8* and (**F**) *nfkb*. The bars represent different treatments. CTL, control; MP, microplastics; TI, thermal increase; MP + TI, microplastics and thermal increase at 24 h. Symbols (*) over the bars indicate statistical differences between different treatments. One-way ANOVA followed by Tukey’s test (** *p* < 0.01, *** *p* < 0.001, **** *p* < 0.0001). n = 5 per sampling day/treatment.

Foregut: The expression of the immune genes tested in this tissue was significantly impacted by either single or combined treatments, except for *tlr4* (Figure 3B). Levels of mRNA for *tlr2* were significantly upregulated in the intestine of fish exposed to a combination of increased temperature and MPs (Figure 3A) but *myd88*, *il6* and *il8* were only stimulated by high temperature alone, while MPs and MPs in combination with high temperature reduced *il6* expression (Figure 3C–E). The expression for the transcription factor *nfkb* was significantly increased by MPs or temperature, but not by the combination of these two treatments (Figure 3F).

***Harpagifer antarcticus***

**Figure 4:**
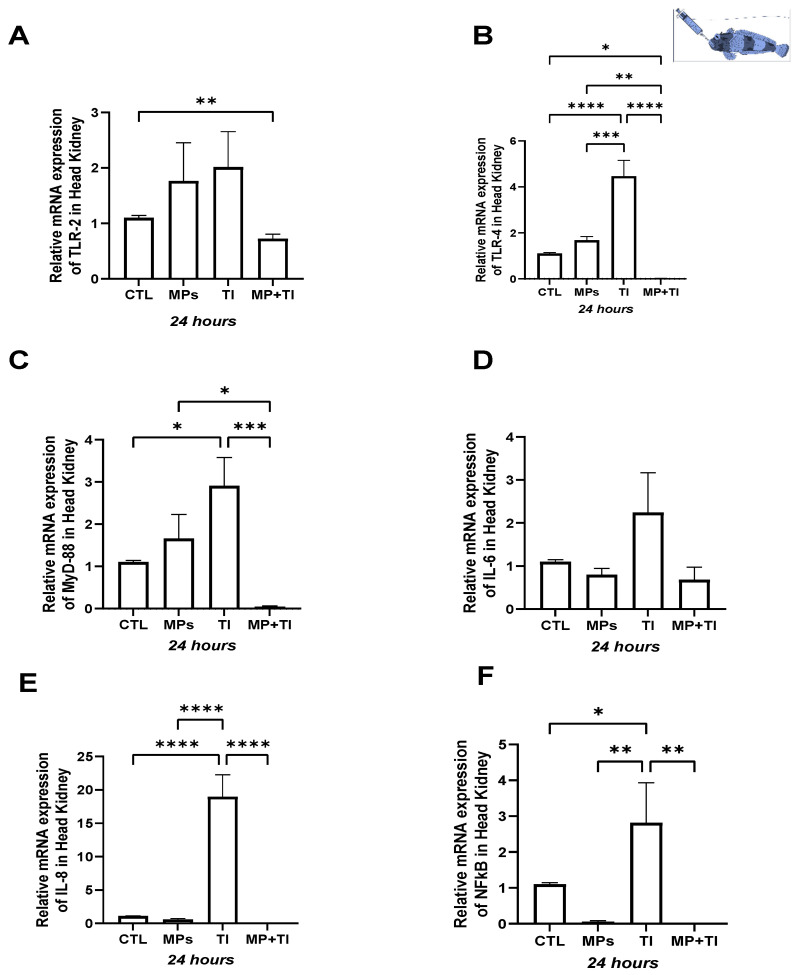
Head kidney Gene Expression of *Harpagifer antarcticus* from the cannula experiment. (**A**) *tlr2*, (**B**) *tlr4*, (**C**) *myd88*, (**D**) *il6*, (**E**) *il8* and (**F**) *nfkb*. The bars represent different treatments. CTL, control; MP, microplastics; TI, thermal increase; MP + TI, microplastics and thermal increase at 24 h. Symbols (*) over the bars indicate statistical differences between different treatments. One-way ANOVA followed by Tukey’s test (* *p* < 0.05, ** *p* < 0.01, *** *p* < 0.001, **** *p* < 0.0001). n = 5 per sampling day/treatment.

Head kidney: In the head kidney of the Antarctic species *H. antarcticus*, the most striking gene expression changes were seen after exposure to increased temperature alone or the combination of microplastics (MPs) and thermal increase, although the effects were in opposite directions. All target genes show some up-regulation (mostly between 2- and 4-fold) in the high-temperature group, reaching an over 15-fold increase in the case of *il8*, with *il6* being the only target gene where the change was not statistically significant. However, when exposed to both high temperature and MPs, all selected genes, again except for *il6*, showed significant down-regulation compared to the control group. Fish exposed to MPs alone did not show significant changes in the gene expression of any of the target genes relative to control fish (Figure 4A–F).

**Figure 5:**
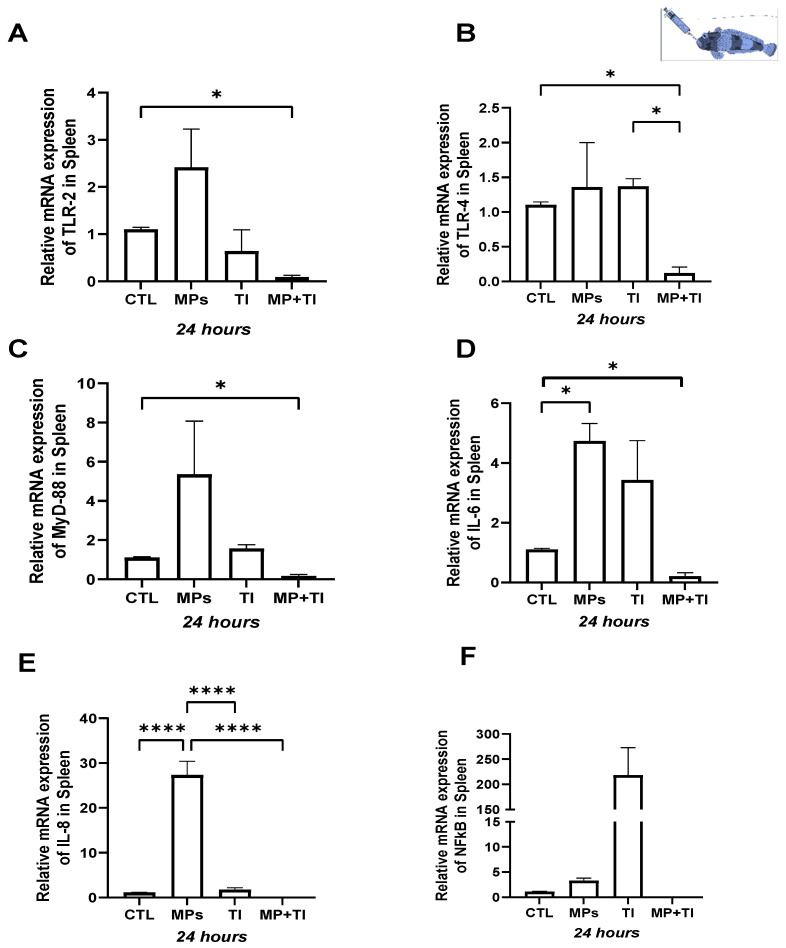
Spleen Gene Expression of *Harpagifer antarcticus* from the cannula Experiment. (**A**) *tlr2*, (**B**) *tlr4*, (**C**) *myd88*, (**D**) *il6*, (**E**) *il8* and (**F**) *nfkb*. The bars represent different treatments. CTL, control; MP, microplastics; TI, thermal increase; MP + TI, microplastics and thermal increase at 24 h. Symbols (*) over the bars indicate statistical differences between different treatments. One-way ANOVA followed by Tukey’s test (* *p* < 0.05, **** *p* < 0.0001). n = 5 per sampling day/treatment.

Spleen: In this tissue, *il6* and *il8* were significantly up-regulated (by ~5- and 25-fold, respectively) in fish exposed to MPs alone, while *tlr2* and *myd88* expression were only slightly but not significantly increased. Exposure to high temperature alone did not result in any changes in expression relative to control fish for any of the target genes. However, when the fish were subjected to a combination of MPs and thermal increase (MPs + TI), all genes, except for *nfkb*, showed significant reductions in transcription levels compared to the control group (Figure 5A–F).

**Figure 6:**
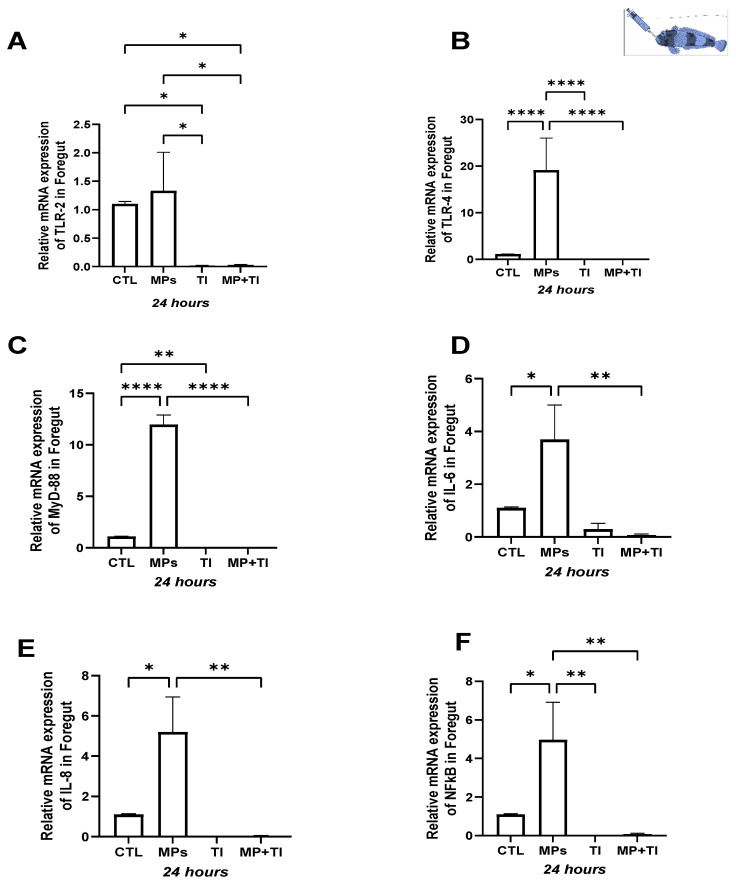
Foregut Expression of *Harpagifer antarcticus* from the cannula Experiment. (**A**) *tlr2*, (**B**) *tlr4*, (**C**) *myd88*, (**D**) *il6*, (**E**) *il8* and (**F**) *nfkb*. The bars represent different treatments. CTL, control; MP, microplastics; TI, thermal increase; MP + TI, microplastics and thermal increase at 24 h. Symbols (*) over the bars indicate statistical differences between different treatments. One-way ANOVA followed by Tukey’s test (* *p* < 0.05, ** *p* < 0.01, **** *p* < 0.0001). n = 5 per sampling day/treatment.

Foregut: In this evaluated tissue, the responses were more marked. The expression of *tlr2* was significantly reduced when the fish were subjected to the thermal increase only, or to the combination of elevated temperature and MPs (Figure 6A). In contrast, exposure to MP strongly induced the expression of *tlr4*, *myd88*, *il6*, *il8*, and *nfkb* (Figure 6B–F). However, no upregulation occurred when the fish faced heat stress alone or in combination with MPs; instead, clear reductions were observed in all the genes evaluated in this tissue (Figure 6A–F).

(B) **Exposure to waterborne MPs**

***Harpagifer bispinis***

**Figure 7:**
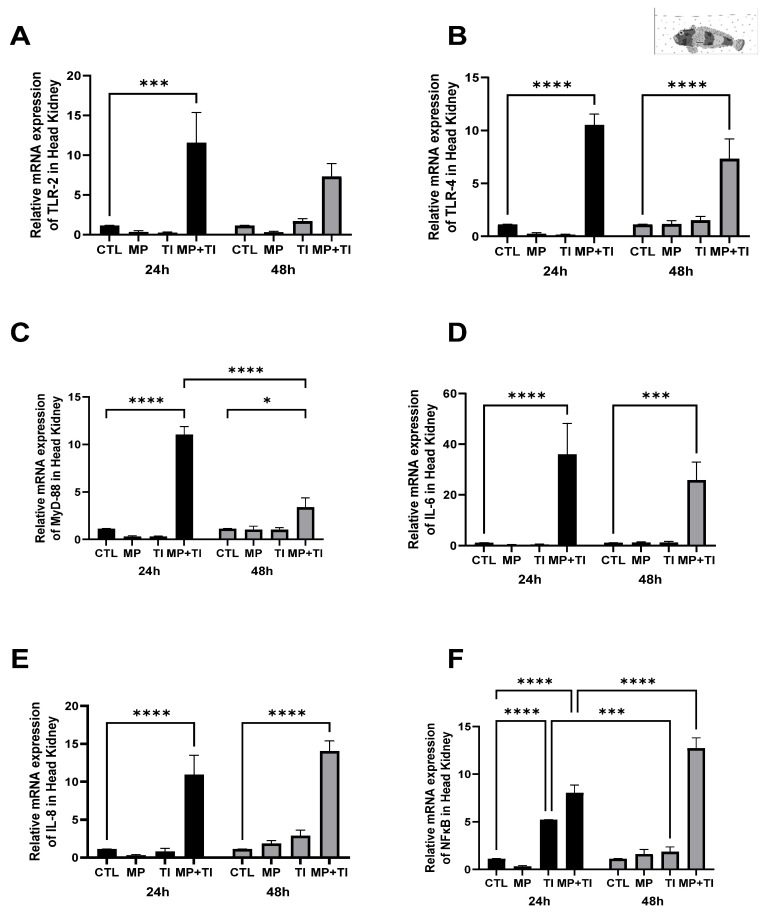
Head kidney Expression of *Harpagifer bispinis* from the bath experiment (**A**) *tlr2*, (**B**) *tlr4*, (**C**) *myd88*, (**D**) *il6*, (**E**) *il8* and (**F**) *nfkb*. The bars represent different treatments. CTL, control; MP, microplastics; TI, thermal increase; MP + TI, microplastics and thermal increase at two exposure times: 24 and 48 h. Symbols (*) over the bars indicate statistical differences between different treatments. Two-way ANOVA followed by Tukey’s test (* *p* < 0.05, *** *p* < 0.001, **** *p* < 0.0001). n = 5 per sampling day/treatment.

Head kidney: In this immune tissue, all target genes were clearly up-regulated in fish simultaneously exposed to heat increase and microplastics, both at 24 and 48 h (Figure 7A–F). This increase was substantial, with increases ranging from 10- to 20-fold in relation to the control levels and even reaching almost 60-fold difference in the case of *il6*. No effect of either MPs alone or temperature alone was observed after 24 h of exposure, except for *nfkb*, which was significantly over-expressed in fish at warm temperature but reduced to control levels after 48 h.

**Figure 8:**
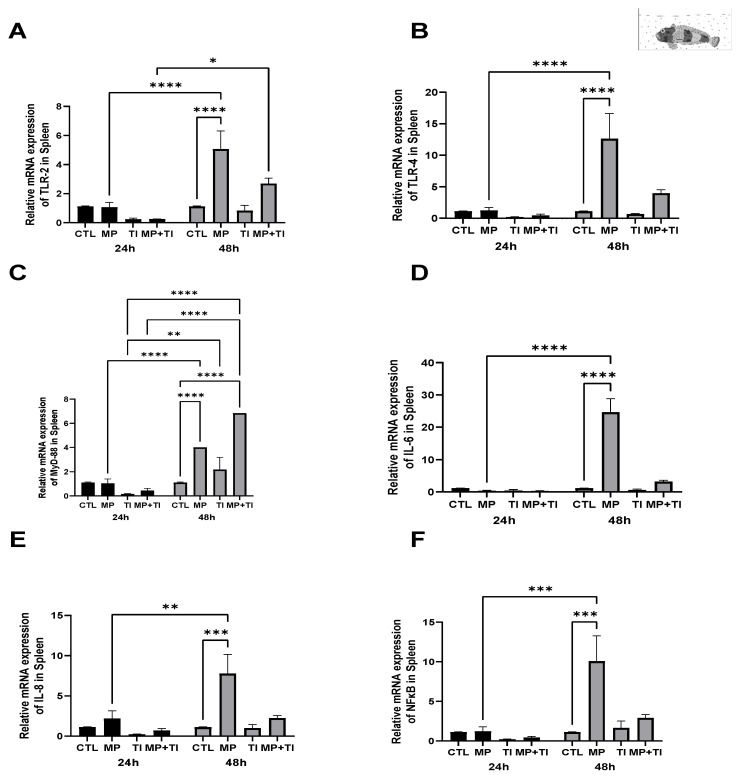
Spleen Expression of *Harpagifer bispinis* from the bath experiment. (**A**) *tlr2*, (**B**) *tlr4*, (**C**) *myd88*, (**D**) *il6*, (**E**) *il8* and (**F**) *nfkb*. The bars represent different treatments. CTL, control; MP, microplastics; TI, thermal increase; MP + TI, microplastics and thermal increase at two exposure times: 24 and 48 h. Symbols (*) over the bars indicate statistical differences between different treatments. Two-way ANOVA followed by Tukey’s test (* *p* < 0.05, ** *p* < 0.01, *** *p* < 0.001 and **** *p* < <0.0001). n = 5 per sampling day/per treatment.

Spleen: In this tissue, *tlr2* expression was slightly lower in the fish exposed to the increased temperature and the combination of both stressors after 24 h of exposure. However, at 48 h a significant increase was observed in fish exposed to microplastics, while just a slight increase occurred when MPs were in combination with temperature (Figure 8A). Although no significant changes were detected at 24 h, at 48 h there was a large increase in *tlr4* expression in fish exposed to MPs alone and a minor up-regulation in the combination with the thermal increase (Figure 8B). In the case of *myd88*, this gene was also more affected at 48 h, rising both in fish exposed only to MPs and to the combination with temperature (Figure 8C). *il6*, *il8* and *nfkb* increased significantly only with MPs at 48 h (Figure 8D–F).

**Figure 9:**
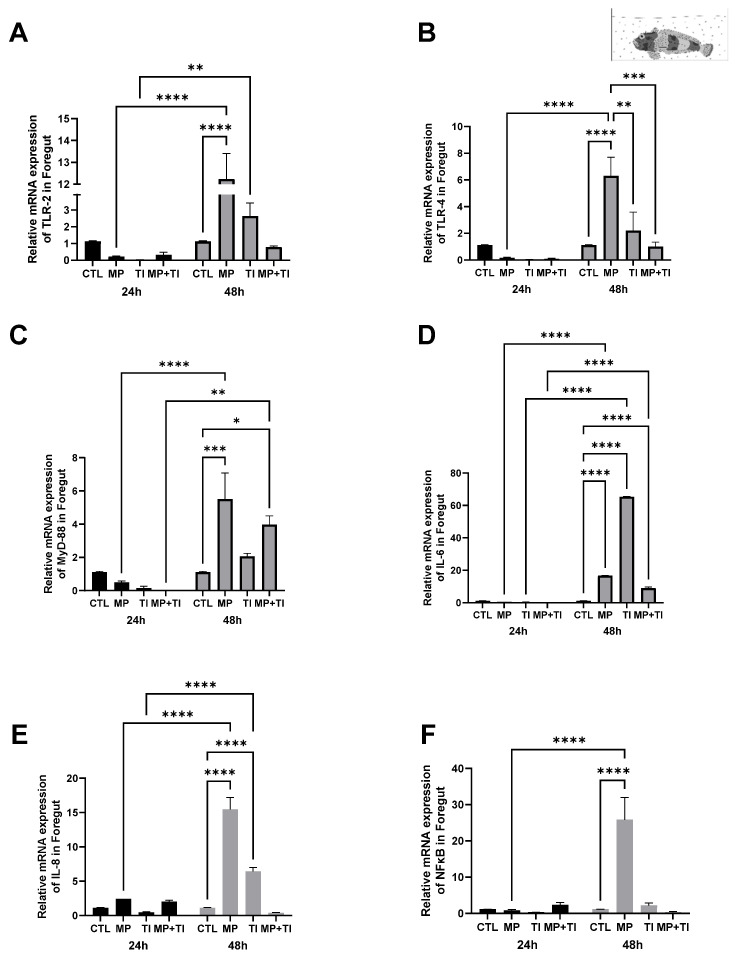
Foregut Expression of *Harpagifer bispinis* from the bath experiment (**A**) *tlr2*, (**B**) *tlr4*, (**C**) *myd88*, (**D**) *il6*, (**E**) *il8* and (**F**) *nfkb*. The bars represent different treatments. CTL, control; MP, microplastics; TI, thermal increase; MP + TI, microplastics and thermal increase at two exposure times: 24 and 48 h. Symbols (*) over the bars indicate statistical differences between different treatments. Two-way ANOVA followed by Tukey’s test (* *p* < 0.05, ** *p* < 0.01, *** *p* < 0.001 and **** *p* < 0.0001). n = 5 per sampling day/per treatment.

Foregut: In intestinal tissue, impacts in gene expression were only evident after 48 h of treatment. While no significant changes occurred for any treatment or target gene at the 24 h sampling, at 48 h transcripts for *tlr2* and *tlr4* increased significantly in subantarctic fishes exposed MPs alone and less in those at elevated temperature alone. The combination of factors showed no difference in relation to the 24h or the 48 h control groups. (Figure 9A,B). A similar profile was observed for *il8* and *nfkb*, which were affected mainly by MPs and only slightly by the temperature increase, while the combinations of both produced no effect (Figure 9E,F). The *myd88* showed a significant increase under exposure to MPs, and the combination of both conditions, but also rose slightly under temperature increase alone (Figure 9C), while *il6* (Figure 9D) was upregulated in all treatments for 48 h (Figure 9F).

***Harpagifer antarcticus***

**Figure 10:**
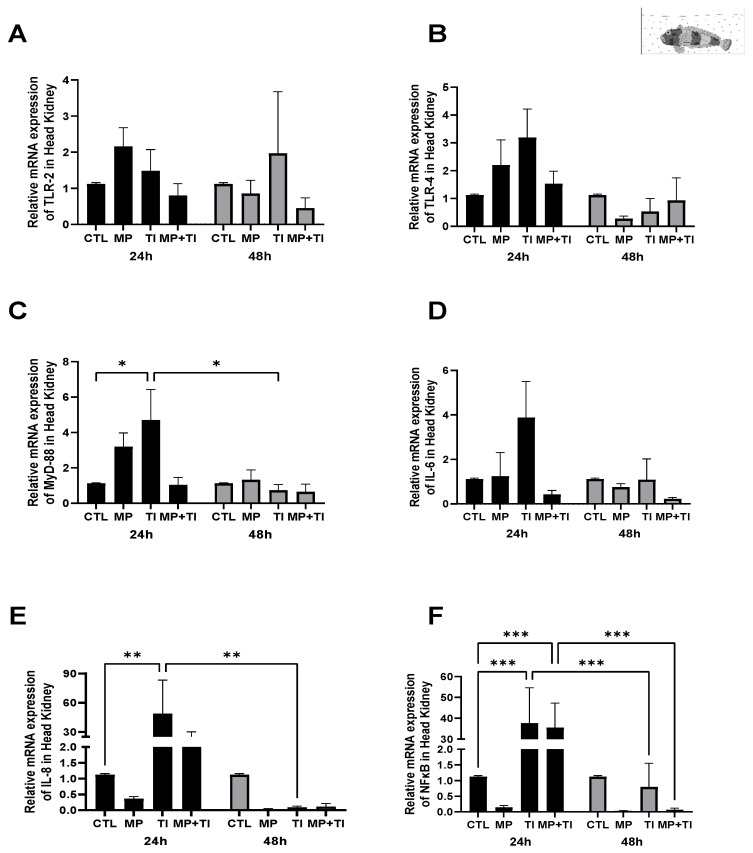
Head kidney Expression of *Harpagifer antarcticus* from the bath experiment. (**A**) *tlr2*, (**B**) *tlr4*, (**C**) *myd88*, (**D**) *il6*, (**E**) *il8* and (**F**) *nfkb*. The bars represent different treatments. CTL, control; MP, microplastics; TI, thermal increase; MP + TI, microplastics and thermal increase at two exposure times: 24 and 48 h. Symbols (*) over the bars indicate statistical differences between different treatments. Two-way ANOVA followed by Tukey’s test (* *p* < 0.05, ** *p* < 0.01, *** *p* < 0.001). n = 5 per sampling day/treatment.

Head kidney: The expression of *tlr2* and *tlr4* showed slight but non-significant variations at 24 h in Antarctic fish exposed separately to microplastics and thermal rise (Figure 10A,B). Expression of *myd88* showed a rise after 24 h, especially in fish subjected to the increase in temperature, and less in those exposed to MPs, compared to the control group. The combined treatment did not produce changes in expression. All had subsided after 48h (Figure 10C). Similarly, *il6* expression increased at 24 h only with the thermal increase to 5 °C. However, this effect disappeared after 48h (Figure 10D). Both *IL8* and *nfkb* showed significant increases at 24 h when exposed to increased temperatures, and in the combined treatment, the latter is only in the case of *nfkb*, reaching over 30-fold difference in relation to the control fish. However, both genes returned to control conditions after 48 h, regardless of the treatments (Figure 10E,F).

**Figure 11:**
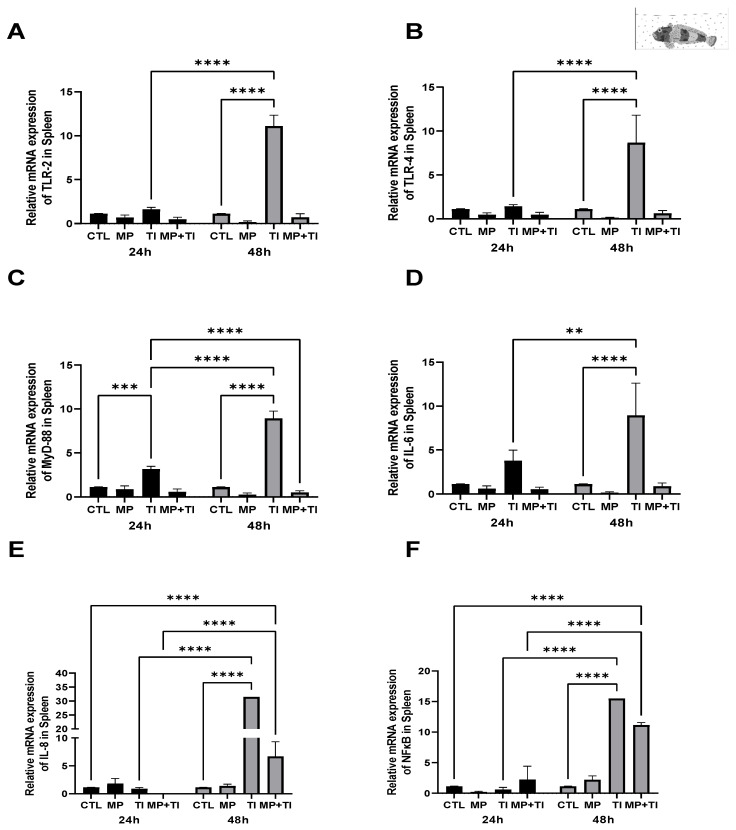
Spleen Expression of *Harpagifer antarcticus* from the bath experiment. (**A**) *tlr2*, (**B**) *tlr4*, (**C**) *myd88*, (**D**) *il6*, (**E**) *il8* and (**F**) *nfkb*. The bars represent different treatments. CTL, control; MP, microplastics; TI, thermal increase; MP + TI, microplastics and thermal increase at two exposure times: 24 and 48 h. Symbols (*) over the bars indicate statistical differences between different treatments. Two-way ANOVA followed by Tukey’s test (** *p* < 0.01, *** *p* < 0.001, and **** *p* < 0.0001). n = 5 per sampling day/treatment.

Spleen: In this tissue expression of *tlr2* and *tlr4*, increased significantly in fish exposed to the temperature increase, but only at 48 h (Figure 11A,B). None of the other treatments, including temperature in combination with MPs, had effects on these two genes. In the case of *myd88* and *IL6*, the effects of temperature on expression were already visible after 24 h and became more significant after 48 h (Figure 11B,C). As for the previous genes, there were no other effects from the remaining treatments. However, *il8* and *nfkb* were significantly affected when the fish faced the thermal increase but also showed an incipient effect when elevated temperature was combined with MPs, but only after 48 h (Figure 11E,F).

**Figure 12:**
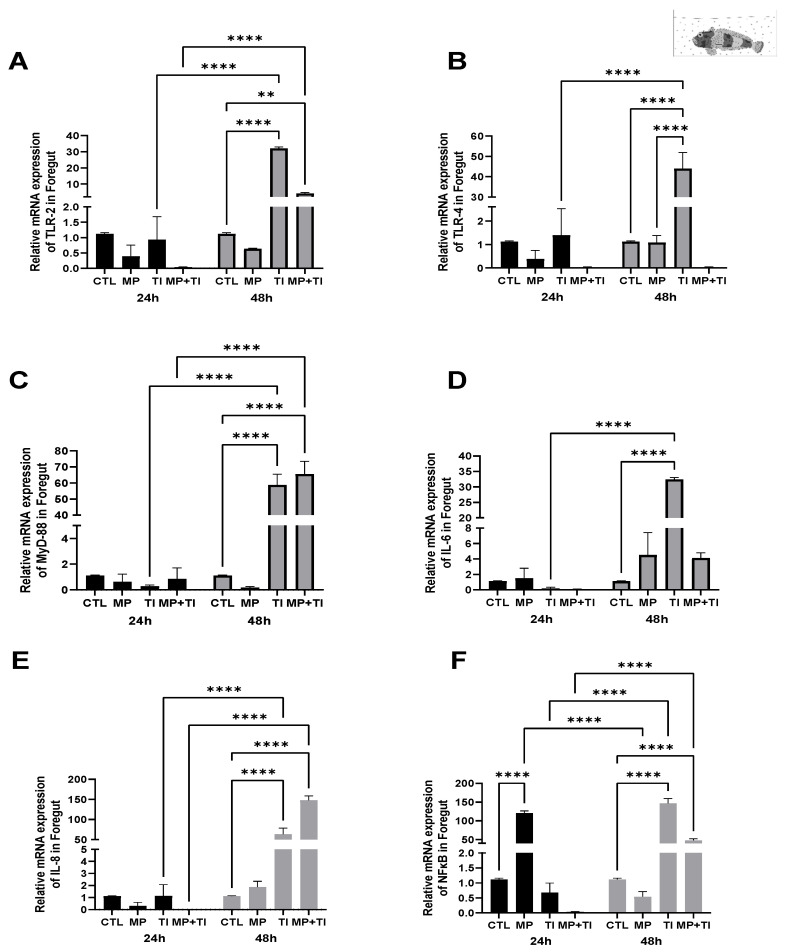
Foregut Expression of *Harpagifer antarcticus* from the Bath Experiment. (**A**) *tlr2*, (**B**) *tlr4*, (**C**) *myd88*, (**D**) *il6*, (**E**) *il8* and (**F**) *nfkb*. The bars represent different treatments. CTL, control; MP, microplastics; TI, thermal increase; MP + TI, microplastics and thermal increase at two exposure times: 24 and 48 h. Symbols (*) over the bars indicate statistical differences between different treatments. Two-way ANOVA followed by Tukey’s test (** *p* < 0.01, **** *p* < 0.0001). n = 5 per sampling day/treatment.

Foregut: Responses of our target genes in the intestinal tract of the Antarctic plunderfish were only evident after 48 h of exposure, except for *nfkb*, which showed a significant increase (over 100-fold) in fish exposed to waterborne MPs within 24 h of treatment. After 48 h, the expression of *tlr2*, *tlr4*, *myd88*, and *il6* was significantly increased (between 30 and 60-fold) in fish exposed to increased temperature and, in the cases of *tlr2* and *myd88*, also in those exposed to the combined treatment (Figure 12A–D). Finally, both *il8* and *nfkb* also increased with thermal increase Expression of *il8* was substantially increased in the high temperature and in the combined treatment groups (reaching about 60- and 140-fold in relation to the control group at the same time point (at 48 h, respectively), and the same occurred for *nfkb* (with similarly high fold differences, about 140 for the high temperature and 60 for the combined treatment). Contrary to the 24 h sampling, no effect of MPs was observed at 48 h in this gene (Figure 12E,F).

## 3. Discussion

This study evaluated the combined effects of PVC microplastic (MP) exposure and increased temperature on two sister notothenioid fish species, *Harpagifer antarcticus* and *Harpagifer bispinis*, using two exposure methods (oral and bath). Gene expression patterns of key immune markers of innate immunity; Toll-like receptors (*tlr2*, *tlr4*), the adaptor protein *myd88*, and the proinflammatory factors *il6*, *il8*, and nfκb, were analyzed in three immunocompetent tissues; the head kidney, spleen, and foregut. When the MPs were gavaged directly into the alimentary canal, the response to MPs was more localized. Particularly in the foregut, showing more acute effects, with significant differences occurring within 24 h. The foregut was also quite responsive to waterborne MPs and to increased temperature, likely due to the warm water and contaminants reaching the intestine via drinking. In this case, the responses to MP exposure only became evident 48 h after the onset of exposure.

The potential immune responses to oral MPs were particularly evident in the foregut of the Antarctic species. This exposure promoted the activation of the (*tlr2*, *myd88*, nfκb) axis, which could lead to significant modulation in the immune response, since they collectively act as a central signaling pathway mediating inflammation and promoting the secretion of proinflammatory cytokines including IL-1β, TNF-α, and *IL6* [51]. Similar effects were observed in Nile tilapia (*Oreochromis niloticus*) exposed to polystyrene microplastics (PS-MP) by [52], who also documented that oral ingestion of PS-MP induced inflammation in liver tissues. This study is the first assessment of acute exposure to PVC-MP in Antarctic and subantarctic fish, and the observed responses are suggestive of a direct interaction between microplastics and the intestinal mucosa. In *H. antarcticus*, the expression of the selected genes in the head kidney showed a little response to MPs but a significant reaction to temperature, whereas in the spleen, the highest response observed resulted from the combined heat and MP treatment.

In tissues such as the spleen and foregut, PVC ingestion decreased gene expression of immune response markers in subantarctic species but evoked little response in the head kidney. Espinosa et al. (2018) [53] evaluated the in vitro effects of PVC-MP and polystyrene MP on head kidney leukocytes of sea bream (*Sparus aurata*) and sea bass (*Dicentrarchus labrax*). After 1 and 24 h of exposure, cell viability decreased, as did innate immune parameters (such as phagocytic activity, respiratory burst, and expression of genes related to inflammation (e.g., IL-1β), oxidative stress, and cell apoptosis). Since IL-IB was activated in the present study, similar physiological responses could also be occurring in *H. antarcticus* and *H. bispinis*. It also remains to be seen if and how many of the MPs reaching the intestine may actually go across the intestinal epithelia to reach immune organs. In this context, it has been reported that MP or nanoplastics (NP) might cause diverse adverse effects on aquatic organisms at multiple biological levels, ranging from single-cell damage to whole-organism impairments, including cytotoxicity, alteration in reproduction, behavioral changes, and oxidative stress [45,46,47,48,49,50,51,52,53,54]. Moreover, exposure to NP has been shown to increase fish susceptibility to nodavirus infection and to reduce antiviral immune responses [54], further underscoring the immunotoxic potential of plastic pollution in aquatic systems [55].

Overall, the fish’s response to increased temperature led to an activation of transcripts for TLRs, *myd88*, and interleukins 6 and 8 in all tissues analyzed in the subantarctic species. However, in the Antarctic species, the same genes were downregulated in the spleen and foregut. This could be related to the fact that temperature is one of the main environmental factors influencing the biochemical, physiological, and behavioral processes of ectothermic marine organisms [56]. Since fish are ectothermic animals, they must maintain a stable and independent metabolic rate, unaffected by water temperature fluctuations, through cellular mechanisms that compensate for these changes [57]. The Antarctic nototheniids appear to have lost part of this cellular stress response [58] and their heat shock proteins do not readily respond to increases in water temperature [59,60]. The current information about their innate immune system, and namely the TLRs and their response cascades, indicates these were only slightly affected by the rapid evolution and adaptation to cold water temperatures [33,34,41,61]. Acute thermal stress events as well as short-term mild warming are known to modify the expression of genes like TLRs, lysozyme, complement factors, and proinflammatory cytokines as part of an acute stress/inflammatory response [61,62,63,64]. However it is important to highlight that the temperature increase used in the experiment is within the usual ranges that the species could experience in its intertidal habitat [65], so these rapid genetic activations or decreases in response to the increase in temperature suggest that the species could have adaptive mechanisms to cope with changes within the thermal range it experiences in its natural habitat [66]. However, when exposed to both stressors, we observed a response characterized by a reduction or inactivation of the expression of immune markers, including Toll-like receptors and interleukins, as well as *nfκb*. These results indicate that the combination of stressors negatively impacts both species in this component of the immune system, but does result in activation of other stress pathways such as oxidative stress [45], promoting tissue damage or activating immune responses and pro-apoptotic reactions similar to those observed by Saravia et al. (2024) [61] when fish are exposed to increased temperature and immunostimulants that mimic pathogens. In general, the presence of various stressors, irritants such as MP, or injuries can provide the basis for initiating an acute reaction (inflammation), which can develop within minutes to hours (24 or 48 h in this study). Furthermore, depending on the timing, environmental stress, the trigger, the anatomical site (inflammation may affect any tissue), or the severity of the inflammation produced, the type of cells and the mediators involved will vary [62].

The results show that, when fish were completely submerged in water containing microplastics and exposed to high temperatures, the response was more global, with complex responses observed in the spleen, foregut, and head kidney, especially in *H. bispinis*. In this context, aqueous exposure appears to provoke a more diffuse response at the systemic level, as *H. bispinis* showed increased immune gene expression, particularly in response to microplastics in the spleen and intestine, as well as to combined stressors in the head kidney. With a more pronounced activation pattern when both stressors were combined in the foregut, this inflammatory response could reflect the greater incorporation of microplastics into tissues as a result of whole-body exposure [67,68]. As temperature increases, so do ingestion and drinking rates [69,70], potentially allowing microplastics to have greater exposure to the gut as well as direct exposure of the gills and skin. In zebrafish exposed to MP-PE and MP-PS for twenty days, alterations in the expression of immune system genes (*MHCII*, *hsp60*, *hsp70*), lymphocyte activation, and downregulation of genes related to epithelial integrity and lipid metabolism were found in gills and intestinal epithelium [70]. However, in *H. antarcticus*, it was observed that exposure to increased temperature led to increased expression of immunological genes in the three tissues analyzed at both times (24 and 48 h). Although fish are known to cope with single stressors that disrupt their homeostasis [71], these changes become more pronounced when multiple stressors are present, often creating synergistic effects that increase the stressful impact. However, when subantarctic and Antarctic fish are faced with both stressors in combination, the increase is observed only in the head kidney (for all genes) and foregut, respectively. Similar effects were observed by Saravia et al. (2024) [61], who evaluated immune response genes during in vitro and ex vivo experiments, with combinations of thermal increase and immunostimulatory agents (LPS and POLY I:C). This implies that transcripts increase or decrease, since the immune response is linked to fish health and depends on the interrelationship of some components of the fish with the environment in which they live, such as the species, the presence of pathogens, the nutritional status of the animal, and the environment [72]. The latter may be the most critical component, as environmental quality significantly influences the physiological well-being of fish, including farmed species, as well as feeding regimes, growth rates, and the ability to maintain innate and acquired immunity and resilience [56]. The observed interspecific differences clearly reflect the ecological and evolutionary adaptations of each species. *H. antarcticus*, adapted to stable, cold environments, showed transcriptional inhibition of proinflammatory genes when faced with both variables simultaneously, which could be interpreted as an “immune economy” strategy to avoid unnecessary responses in unpredictable environments [73,74,75]. Furthermore, the combination of microplastics and temperature does not always produce an additive or synergistic response, which could indicate a form of multiple stress-induced immunosuppression or a cellular desensitization phenomenon. This pattern has been reported in fish under sustained thermal stress, where the initial expression of immune genes is reduced at later stages as a protective mechanism against autoimmune damage [61]. In contrast, *H. bispinis*, which inhabits more temperate and dynamic zones, showed greater susceptibility to thermal and microplastic exposure, suggesting a lower adaptive capacity to these stressors, especially when combined. This behavior is also consistent with reports from studies on other subantarctic species, which tend to be more susceptible to rapid environmental changes [76]. Together, the immune genes *tlr2*, *tlr4*, *myd88*, nfκb, *il6*, and *il8* are essential for the immune defense of fish in Antarctic and subantarctic regions, not only against pathogens, but also against stressors such as rising temperatures and exposure to pollutants, including microplastics. A detailed understanding of the function of these genes in the context of these environmental challenges is key to predicting the impact of global changes on the health and survival of these species.

## 4. Materials and Methods

The experimental procedures and sample handling complied with ethical guidelines that regulate the use of animals in the laboratory, established by the National Commission for Scientific and Technological Research (CONICYT, Chile), FONDAP-IDEAL 15150003, Millennium Institute Biodiversity of Antarctic and Sub-Antarctic Ecosystems, BASE, and the Universidad Austral de Chile.

**Animals and Experimental Setup**

Adult specimens of *H. antarcticus* (n = 60, mean body mass = 14.5 g, mean body length 9.8 cm) and *H. bispinis* (n = 60, mean body mass = 12.8 g, mean body length = 8.9 cm) were collected manually by turning over rocks in the intertidal zone of Fildes Bay (King George Island, Antarctica) and Punta Arenas (Puerto Bulnes, Chile), respectively. The collected fish were acclimatized for at least 72 h in seawater collected at the sampling site at 2 °C, 33 PSU (King George Island, Antarctica), and 8 °C, 33 PSU (Punta Arenas, Chile), respectively, as these temperatures reflect the natural conditions at the sites of sampling and season (austral summer). The fish were maintained under natural summer photoperiod and constant aeration, and they were fed ad libitum with their natural amphipod diet, collected from the study sites [76]. After an acclimation period, one group of fish was kept at the control temperature, while the other group was subjected to temperature increase following an established protocol [45,46,47,48,49,50,51,52,53,54,55,56,57,58,59,60,61,62,63,64,65,66,67,68,69,70,71], reaching 5 °C for *H. antarcticus* and 12 °C for *H. bispinis*. Once the target temperature was stabilized, individuals from the control and high-temperature groups were randomly distributed into experimental 2 L glass aquaria (without water flow but with constant aeration) and placed in large temperature-controlled water baths (2–5 °C and 8–12 °C). For each species and each MPs exposure method, four experimental groups (n = 5 per group) were established: (i) a control group at 2 °C, which is the temperature with the natural acclimatization of the Antarctic species, or at 8° for the subantarctic species; (ii) a group exposed to PVC microplastic at the same temperature (2 °C or 8 °C); (iii) a group with a thermal increase at 5 °C for *H. antarcticus* or 12 °C for *H. bispinis*; and (iv) a group exposed to both PVC microplastic and elevated temperature (5 °C or 12 °C). The experimental temperature was controlled by placing the aquaria in thermostatic water baths, each equipped with an Osake T20 temperature controller (Omron Corporation, Tokyo, Japan) connected to a coarse aquarium heater. An Astro 2000 submersible pump (Zhejiang Astro Mechanical & Electrical Co., Ltd., Wenzhou, China) homogenized the water. The fish were maintained at the corresponding temperatures for each condition for 24 (cannulation experiment) or 24–48 h (bath or aqueous exposure experiment), depending on the treatment. Fish were not fed during experimental periods.

**Microplastics and MP exposure**

In the present study, spherical commercial PVC microplastics (MPs; 3–10 μm in diameter; Sigma-Aldrich^®^ (Product No. 189588, Polyvinyl chloride, St. Louis, MO, USA)), were used, following the protocol described by [45]. The dose used was 20 µg for direct delivery into the stomach by cannula and 2000 µg/L for external exposure in a bath; the dosing was chosen according to the results reported by [77]. A concentration of 200 µg/L or 2000 µg/L of PVC microplastics was selected for this study, based on [30,45], who reported no lethality at this dose in fish. Although this concentration exceeds current environmental levels (estimated at ~0.01 g/L), it was intentionally chosen to elicit measurable physiological responses without causing mortality.

**(A) Cannula delivery of MP (oral):** The cannula technique used the same specimens and experimental procedures as those in [45]. Fish randomly collected from the acclimatization tank were gavaged with 20 µg of PVC of MPs directly into the stomach using physiological saline (0.9% NaCl), with volumes of 50 μL for *H. bispinis* and 100 μL for *H. antarcticus*. These volumes were adjusted according to species-specific body size, as *H. bispinis* is smaller than *H. antarcticus*, requiring a lower gavage volume to avoid gastric distension and ensure animal welfare. For the control group, fish were gavaged with a physiological saline solution alone. Upon completion of gavage, fish from each treatment group were allocated to a separate 2 L glass aquarium.

**(B) Bath exposure of MP (aqueous)**: Fish selected from the acclimatization tanks were randomly distributed, as described above, into either control aquaria or aquaria containing waterborne MPs. To prepare the exposure, MPs were mixed in physiological saline solution and delivered to the tank at a concentration of 2000 μg/L. An identical volume of physiological saline solution was added to the control tanks. To ensure that the exposure dose remained stable throughout the experimental period, water was renewed at each experimental time point (24 h and 48 h), and the corresponding MP suspension was replenished to restore the target concentration. The specimens were kept at the corresponding temperatures for each condition for 48 h, which was the duration of the experiment.

**Fish sampling procedure**

At the end of each experimental period, fish were sampled following the protocol described by Vargas-Chacoff et al. (2021) [71,78]. The specimens were swiftly collected and anesthetized with a lethal dose of 2-phenoxyethanol (1 mL/L, Sigma-Aldrich-Fluka, St. Louis, MO, USA), weighed, and sacrificed by severing the cervical spinal cord. The fish were then dissected, and the head kidney, spleen, and foregut were extracted from each specimen. These tissues were snap-frozen in liquid nitrogen and stored at −80 °C until analysis.

**Gene expression analysis.**

**Total RNA extraction**

Total RNA was extracted from the head kidney, spleen, and foregut using TRIzol reagent (Sigma, St. Louis, MO, USA) following the manufacturer’s instructions and stored at −80 °C. The samples were treated with amplification-grade DNase I (1 U/μg RNA, Invitrogen) and subsequently quantified using a NanoDrop spectrophotometer (NanoDrop Technologies, Wilmington, DE, USA). Finally, total RNA (2 μg) was used in reverse transcription to synthesize cDNA, using MMLV-RT reverse transcriptase (Promega, Madison, WI, USA) and the oligo-dT primer (Invitrogen, Waltham, MA, USA) according to standard procedures.

**qRT-PCR analysis of gene expression**

The cDNA obtained was diluted to 100 ng/μL to be used as a template to amplify the gene transcripts by real-time PCR of toll-like receptor 2 (*tlr2*), toll-like receptor 4 (*tlr4*), myeloid differentiation factor 88 (*myd88*), nuclear factor kappa b (*nfkb*), interleukin-6 (*il6*), interleukin-8 (*il8*), and β-actin as housekeeping reference (Table 1). The real-time PCR was conducted in an AriaMx qPCR System thermocycler (Agilent Technologies, Santa Clara, CA, USA) using Brilliant II SYBR Green qPCR Mastermix (Stratagene, La Jolla, CA, USA). Each reaction was performed in duplicate, with a total volume of 14 μL, consisting of 6 μL of SYBR Green, 2 μL of cDNA, 1.08 μL of primer mix, and 4.92 μL of molecular-grade water. Amplification was carried out according to a published protocol 95 °C for 10 min to activate the polymerase, followed by 40 cycles at 90 °C for 10 s, 60 °C for 15 s, and 72 °C for 15 s. A dissociation curve was performed at the end of each reaction to verify that a single product was amplified and the absence of primer dimers. The expression levels were analyzed using the comparative Ct method (2^−ΔΔCT^) [79]. The data are presented as the fold change in gene expression normalized to an endogenous reference gene (β-actin) relative to the treatment control. PCR efficiencies were calculated by linear regression analysis of sample data using LinRegPCR (Microsoft Excel software 365, version 2508) [80] from the serial dilutions, where Log dilution was plotted against DCT (threshold cycle number).

**Statistical Analysis**

Linear categorical variable models were applied to evaluate the response of each treatment for all head kidney, spleen, and foregut parameters analyzed in both species. A One-way and Two-way ANOVA analysis of variance was used for the cannula by using different treatments, i.e., control (CTL), microplastics (MPs), thermal increased (TI), and microplastics, thermal increased (MP + TI), and bath experiments (different treatments CTL, MP, TI and MP + TI and exposure time, i.e., 24 and 48 h), respectively. Post hoc Tukey tests were performed to evaluate a posteriori difference in relative gene expression between groups. The assumptions of normality, independence, and homogeneity of the residuals for the variances between groups were also tested using a Shapiro–Wilk test and a Levene test. All statistical analyses and graphs presented were performed using GraphPad Prism 9 software. 

## 5. Conclusions

The present work utilized two microplastic exposure methods, “oral and aqueous (bath),” to describe the immune response in two sister species, the Antarctic *Harpagifer antarcticus* and the subantarctic *Harpagifer bispinis*. We found clear patterns of gene expression and differences between treatments when *H. antarcticus* and *H. bispinis* were exposed independently to MPs or thermal increase (TI) in the cannula experiment. When exposed to MPs or TI alone, they showed up-regulation of transcripts, whereas when exposed to MP + TI, there was down-regulation. However, transcript expression of the target innate immunity genes in aqueous increased when both stressors were combined, mainly after 48 h of exposure time. These results highlight the importance of evaluating the effects of multiple stressors at different levels, independently or in combination, and whether these species will have the capacity to adapt or survive under these conditions, especially in waters where temperature and plastic pollution, particularly PVC-MPs, which are widely used, are present.

## Figures and Tables

**Table 1:** Primer sequences for the immune system used in experiments.

Primer	Nucleotide Sequences (5′→3′)	Efficiency (%) Head Kidney	Efficiency (%) Spleen	Efficiency (%) Foregut	Reference
Toll-like receptor-2 Fw	ATGGAGCTGTTGACCAACCT	102.9	102.5	102.3	Saravia et al. (2022) [35]
Toll-like receptor-2 Rv	GTCTGTTACCGTGGGAACAAG				
Toll-like receptor-4 Fw	CGTGCTCTTCCCTACATGCT	101.6	101.7	101.8	Saravia et al. (2022) [35]
Toll-like receptor-4 Rv	GCTGTTGGGCTGTGATGTCT				
Myeloid differentiation factor 88 Fw	ACTTCCCAAAACATGGCGTG	100.1	100.2	100.3	(Martínez et al., 2018) [75]
Myeloid differentiation factor 88 Rv	ACCGTGTTCTTCGGGTTCAG				
Nuclear factor-κB Fw	GAAGAAGATGGCGGGAGCTA	105.5	102.6	101.7	Saravia et al. (2022) [35]
Nuclear factor-κB Rv	TGATGTCGACTGGAGGAATGTAG				
Interleukin-6 Fw	TTCTCAGGCAAGTGGAGAAGGAGT	102.9	101.1	101.5	Saravia et al. (2022) [35]
Interleukin-6 Rv	CACCTGACCAGGGTTCCTCATTTT				
Interleukin-8 Fw	GTGAAGGGATGAGTCTGAGAAGTC	102.9	101.1	101.5	Saravia et al. (2022) [35]
Interleukin-8 Rv	TCACAGTGGGAGTTGGCAGAA				
β-actin Fw	AGGTCATCACCATCGGAAACGA	102.11	101.1	101.5	Saravia et al. (2022) [35]
β-actin Rv	ACAGCACGGTGTTGGCGTACA				

## Data Availability

The original data presented in the study are openly available in https://doi.org/10.5281/zenodo.17086001.
